# Effect of Plant Extracts Formulated in Different Ointment Bases on MDR Strains

**DOI:** 10.4103/0250-474X.70494

**Published:** 2010

**Authors:** Pallavi L. Pawar, Bela M. Nabar

**Affiliations:** Department of Microbiology, Smt. Chandibai Himathmal Mansukhani College, Ulhasnagar - 421 003, India

**Keywords:** *Aloe vera*, *Eucalyptus globulus*, *Ficus infectoria*, *Ficus religiosa*, multi-drug resistant strains, *Piper betel*, *Pseudomonas*

## Abstract

Extracts of *Aloe vera* whole plant, *Eucalyptus globulus* leaves, *Ficus infectoria* bark, *Ficus religiosa* bark and *Piper betel* leaves were studied for antibacterial activity on resistant and sensitive strains, isolated from skin and soft tissue infections. A combination of hot alcoholic extracts of *Ficus infectoria*, *Ficus religiosa* and *Piper* betel were found to be more effective against all the isolates. The combined extract was formulated in different ointment bases such as polyethylene glycol, gelatin, sodium alginate, carbopol, cream base and honey. These were then evaluated to find a suitable base for preparation of an ointment. *In vitro* study of the release of antimicrobials and kill-time studies of the herbal ointments was carried out against multi-drug resistant isolate of *Pseudomonas*. The ointment showed bactericidal activity within 2 h against the resistant strain of *Pseudomonas* spp.

Multi-drug resistance among many bacterial species has been reported to be on the increase due to inappropriate or widespread use of antimicrobials[1]. Resistance to antimicrobials has been observed in various parts of the world. During 1997-1998, *Staphylococcus aureus* was found to be the causative agent in most of the skin and soft tissue infections, and among these infections methicillin resistant *Staphylococcus aureus* (MRSA) was the major causative agent[2]. Vancomycin resistant *Enterococcus faocium* has also shown a progressive increase in various infections[3]. Drug resistant strains are causing severe problems in many infections including skin infections such as carbuncles, folliculitis, impetigo, and burn wound sepsis. Antimicrobial resistance prolongs the duration of hospitalization, thereby increasing the cost of patient care.

Globally medicinal plants have been used since ancient times in one form or the other. *Aloe vera* gel[4], neem[5] and eucalyptus oil[6] have been reported to show antimicrobial activity[6]. The aim of the project was to develop an herbal ointment for controlling skin and soft tissue infections due to multi-drug resistant strains.

Five commonly available medicinal plants were selected for the study; they were procured from Kashele Forest Academy, Karjat, India. They were authenticated by the Department of botany, Smt. CHM College for the identification of the plant species before employing them for the study. The plants collected were whole plant of *Aloe vera, Eucalyptus globulus (Nilgiri)* leaves, *Ficus infectoria (Pakar)* bark, *Ficus religiosa (Pipal)* bark, and *Piper betel (Betel)* leaves. The extracts were prepared with ethyl alcohol of analytical grade and distilled water for aqueous extract preparation.

The plant parts were extracted using ethanol and water. Hot alcoholic extract (HAE) was prepared using a Soxhlet extractor. For hot water extract (HWE) 30 g of powder with 300 ml of water was heated in a water bath maintained at 100° till the volume was reduced to one fourth of the original volume[7]. Cold alcoholic extract (CAE) was prepared with 300 ml of ethyl alcohol added to 30 g of powder and shaken for 72 h at room temperatures. For the preparation of 50% cold alcoholic extract (50% CAE) 30 g of powder was taken and to that 150 ml alcohol and 150 ml water was added. This was kept under shaker conditions for 72 h. All the extracts were dried at 50°[7].

Swabs were collected from patients suffering from skin infections like burn wound sepsis, carbuncles, cellulitis, folliculitis, furuncles and impetigo infections. In case of hospital samples transport media i.e. Stuart’s Transport media was used[8]. The sample collection was in presence of the doctor and approvals of their IEC were obtained.

Samples collected were streaked on nutrient agar. According to the Gram’s character and other biochemical reactions, the isolates were identified with the help of Bergey’s Manual[9]. The isolates obtained were checked for their sensitivity to various antibiotics currently used for treating various skin infections by Kirby Bauer disc method[10]. The antibiotics of different groups were used for the study and included aminoglycosides (amikacin, gentamycin, kanamycin, streptomycin, tobramycin), β-lactams (ampicillin, penicillin), chloramphenicol, glycopeptide (vancomycin), lincosamides (clindamycin), macrolides (erythromycin), quinolones (nalidixic Acid, ciprofloxacin), sulfonamides (sulphadiazine), and tetracycline.

Agar ditch method was used for the initial screening of the antibacterial activity of the plant extracts[11]. 0.5 g of extract in 10 ml nutrient agar was used (5% extract). Minimum inhibitory concentration (MIC) of the individual plant extracts was determined by plate dilution technique[12]. Combination of the active extracts of *pakar, pipal* and *betel* were used in the combination form for further study.

Ten different bases were screened for its efficacy for formulation of an ointment[13]. Bases selected were water soluble[14], water washable bases[14], hydrophilic bases[14], gel bases (Aloe gel[15], gelatin base[16], sodium alginate base, Carbopol gel base[14], petroleum jelly[17], coconut oil[18] and honey[19].

Antibacterial activity of the ointments was checked by agar cup method[20]. Kill time studies of the bases and the formulated ointment was carried out[21]. The ointment was subjected to bactericidal time efficacy (contact time period). This method assesses the bactericidal effect of the ointments under study. The organisms are maintained in contact with the ointment and its bactericidal effect was monitored at regular time intervals ranging from 0 to 24 h at an interval of 1 h.

Stability studies of the active ointments were carried out for parameters like pH, colour, odour, antibacterial activity and sterility[14]. The different ointments prepared were checked for stability by standard method. The ointment was kept at 4°, 27°, 37° and 45° for 4 weeks. Each week the ointment was checked for its colour, smell, odour, pH, texture and its antibacterial activity.

All the individual extracts and their combinations were tested for toxicity by topical application in Sprague Dawley rats[22]. This test is designed to measure the potential to cause sensitization and it also provides a measure of irritancy potential of the ointments. The rats were Sprague Dawley, 150 g in weight and the protocol was approved by the IAEC and experiments were carried out in the CPCSEA registered animal house. The dorsal surface of the Sprague Dawley rat was depilated using depilation cream; the rats were anesthetized with anesthetic ether during the procedure. The test material was applied to the depilated dorsal surface of the rat for 72 h. 100 mg of dose was applied each day. Then the skin was observed for 72 h for erythema, edema and necrosis.

The first phase of the project was sample collection from various skin and soft tissue infections. Forty six samples were collected (Table 1). Among the 46 samples collected, 9 samples showed mixed infection and 24 samples had single type of infection. It was observed that *Pseudomonas spp* and *Staphylococcus spp*. were the predominant organisms causing skin infections, each accounting for fifteen, (35%) and thirteen, (30%), respectively, followed up by *Klebsiella spp*. six, (14%), *Enterobacter spp*. five, (12%), *Escherichia coli* (three, 7%) and *Micrococcus spp*. (one, 2%) as shown in fig. 1.

**Fig. 1:**
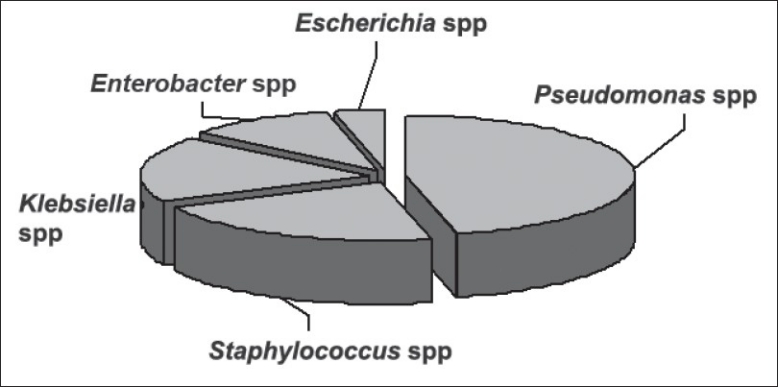
Percentage of different organisms isolated from skin and soft tissue samples. From the skin and soft tissue samples, *Staphylococcus spp* isolated were 30%, *Klebsiella* spp isolated were 14%, *Enterobacter* spp were 12%, *Escherichia* spp were 7% and *Micrococcus spp* were 2%.

**TABLE 1:** SAMPLE COLLECTION DATA

Types of samples	Burn wound sepsis	Acne	Furuncles	Impetigo	Folliculitis	Footsores	Infective eczema	Wounds
Samples	20	5	6	3	1	4	3	4
Total isolates	20	7	5	3	2	2	2	2

According to the antibiotic sensitivity done, more than 50% of the isolates were found to be resistant (fig. 2). Among *Pseudomonas* isolates ten were found to be resistant to major antibiotics. Nine of the *Staphylococcus* was resistant to β-lactams, aminoglycosides, glycopeptides, quinolones, tetracycline and chloramphenicol. From *Klebsiella* spp and *Enterobacter* spp four were resistant. All the three *Escherichia coli* isolates were resistant to major antibiotics.

**Fig. 2:**
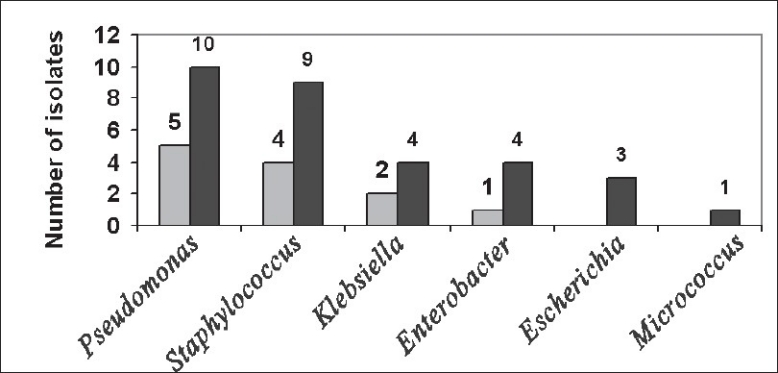
Number of resistant and sensitive strains isolated from the samples. The graph depicts the comparative study of the resistant (■) and sensitive (□) strains isolated from skin and soft tissue samples from patients.

Initial screening of the antibacterial properties of the selected plant extracts was carried out by agar ditch method using 5% concentration of extract. Hot alcoholic extracts of *pakar, pipal, betel*, cold alcoholic extract of *Aloe vera, pakar, betel, nilgiri* and hot aqueous extract of betel were found to possess antibacterial activity against both the resistant and sensitive isolates and also against standard ATCC strains of *Staphylococcus aureus* ATCC no. 2901 and *Pseudomonas aeruginosa* ATCC no. 2862.

MIC of the above extracts (Table 2) was carried out by plate dilution method in the range between 0.5% and 2%. Betel HAE and CAE inhibited the sensitive strains at 0.5% and all the resistant isolates at 1.5%. While *pakar* HAE and CAE inhibited all the isolates at 2% concentration, *Pipal* HAE and *nilgiri* CAE showed activity against all the isolates at 2%. On the basis of MIC results, a combination of hot alcoholic extracts of *pakar* (bark), *pipal* (bark) and *betel* (leaves) was prepared. The *in vitro* antibacterial activity and MIC showed that most of the isolates were inhibited at 0.5% concentration and the rest of the isolates were inhibited at 1.5% concentration.

**TABLE 2:** DETERMINATION OF MINIMUM INHIBITORY CONCENTRATION OF THE PLANT EXTRACTS

Isolates	Extracts	E_R_	K_R_	K_S_	EN_R_	EN_S_	P_R_	P_S_	P. a	S_R_	S_S_	M_R_	S. a
*Nilgiri*	CAE	2%	2%	2%	2%	2%	2%	1.5%	2%	2%	1.5%	1.5%	1.5%
	HAE	2%	2%	1.5%	2%	1.5%	1.5%	1.5%	2%	2%	1.5%	1%	1.5%
*Pakar*	CAE	2%	2%	1.5%	2%	2%	1.5%	1.5%	2%	2%	1.5%	1%	2%
	50% CAE	2%	2%	2%	2%	2%	2%	2%	2%	2%	2%	1.5%	2%
*Pipal*	HAE	2%	2%	2%	2%	2%	1.5%	1.5%	2%	2%	1.5%	1%	1.5%
*Betel*	HAE	1.5%	1.5%	1%	1.5%	1.5%	0.5%	1%	1.5%	1.5%	0.5%	1%	0.5%
	CAE	2%	2%	1.5%	1.5%	2%	0.5%	1.5%	1.5%	1.5%	0.5%	1%	0.5%
*Aloe vera*	CAE 2%	2%	2%	2%	2%	2%	1.5%	2%	2%	1.5%	2%	1.5%

Determination of minimum inhibitory concentration of the hot alcoholic (HAE) and cold alcoholic (CAE) plant extracts against Gram negative resistant *Klebsiella* strains (K_R_), sensitive *Klebsiella* strains (K_s_), resistant *Enterobacter* strains (EN_R_), sensitive *Enterobacter* strains (EN_S_), resistant *Escherichia coli* strains (E_R_), resistant *Pseudomonas* strains (P_R_), sensitive *Pseudomonas aeruginosa* strains (P_S_) and standard *Pseudomonas aeruginosa* ATCC no. 2862. (P. a). The Gram positive resistant *Staphylococcus* strains (S_R_), sensitive *Staphylococcus* strains (S_S_), resistant *Micrococcus* strain (M_R_) and standard *Staphylococcus aureus* ATCC NO.2901(*S. a*).

The individual plant extracts and the combination extract were evaluated for its toxicity on topical application. The extracts exhibited no toxic effect i.e., no erythema, edema and necrosis were observed on topical application. Hence all the extracts were found to be non-toxic in nature and could be used as a topical agent to control infection.

The combination of extracts was further formulated into different bases selected, to prepare a topical ointment. The antibacterial activity of the ointment was checked by agar cup method using resistant strains of *Pseudomonas spp* and *Staphylococcus* spp (Table 3).

**TABLE 3:** ANTIBACTERIAL ACTIVITY OF OINTMENTS BY AGAR CUP METHOD

	Herbal formula combination 1		Base control	
	S_R_	P_R_	S_R_	P_R_
Hydrophilic ointme nt	--	--	--	--
Base A	15	13	--	--
Base B	18	16	--	--
Polyethylene glycol base	28	20	17	15
Aloe gel base	--	--	--	--
Gelatin base	20	17	--	--
Sodium alginate base	19	15	--	--
Carbopol base	17	14	--	--
Petroleum jelly	--	--	--	--
Emulsifying wax base	--	--	--	--
*Taila* (coconut oil)	--	--	--	--
Honey	36	21	22	13

The antibacterial activity of the combination 1 (hot alcoholic extract of *pakar, pipal* and *betel* plant extracts) in different bases was determined against resistant *Staphylococcus* spp. (S_R_) and resistant *Pseudomonas* spp. (P_R_) by Agar cup method and the zone of inhibition was determined in mm.

Hydrophilic ointment, Aloe gel, petroleum jelly and oil base did not exhibit any zone of inhibition towards the resistant organism, due to reduced diffusible property of the formulated ointment. Ointments prepared in sodium alginate base and gelatin base were water-soluble and exhibited good antibacterial activity. However the drawbacks of the bases were that, they were contaminated easily and had no shelf life. Hence the above bases were not considered for further studies.

Kill time studies showed that all the above selected ointments exhibited antibacterial property within 2 h. Base B was effective but was found to be unstable after 2 w, showing change in pH and cracking of the base. Honey and polyethylene glycol bases themselves possessed antibacterial activity, and was observed that the base inhibited the isolate within 7 h and 6 h, respectively.

Base A, polyethylene glycol base, carbopol base and honey showed good antibacterial property. The bases were non-greasy, water-soluble and water washable. These bases were selected for formulation of the plant extract in the form of an ointment.

The ointments showed bactericidal activity within 2 h against resistant strain of *Pseudomonas* spp. Toxicity test carried out on Sprague Dawley rats showed no erythema, edema and necrosis, proving the extracts to be non toxic topically. Thus ointments can be further studied for its wound healing properties *in vivo*.
